# Reliability and clinical utility of spatially constrained estimates of intrinsic functional networks from very short fMRI scans

**DOI:** 10.1002/hbm.26234

**Published:** 2023-02-25

**Authors:** Marlena Duda, Armin Iraji, Judith M. Ford, Kelvin O. Lim, Daniel H. Mathalon, Bryon A. Mueller, Steven G. Potkin, Adrian Preda, Theo G. M. Van Erp, Vince D. Calhoun

**Affiliations:** ^1^ Tri‐Institutional Center for Translational Research in Neuroimaging and Data Science (TReNDS) Georgia State University, Georgia Institute of Technology, and Emory University Atlanta Georgia USA; ^2^ Mental Health Service San Francisco Veterans Affairs Healthcare System San Francisco California USA; ^3^ Department of Psychiatry and Weill Institute for Neurosciences University of California San Francisco San Francisco California USA; ^4^ Department of Psychiatry University of Minnesota Minneapolis Minnesota USA; ^5^ Department of Psychiatry and Human Behavior University of California Irvine Irvine California USA; ^6^ Clinical Translational Neuroscience Laboratory, Department of Psychiatry and Human Behavior University of California Irvine Irvine California USA; ^7^ Center for the Neurobiology of Learning and Memory University of California Irvine Irvine California USA; ^8^ Department of Psychology Georgia State University Atlanta Georgia USA

**Keywords:** resting‐state fMRI (rsfMRI), functional network connectivity (FNC), intrinsic connectivity networks (ICNs), spatially constrained independent components analysis (scICA), schizophrenia

## Abstract

Resting‐state functional network connectivity (rsFNC) has shown utility for identifying characteristic functional brain patterns in individuals with psychiatric and mood disorders, providing a promising avenue for biomarker development. However, several factors have precluded widespread clinical adoption of rsFNC diagnostics, namely a lack of standardized approaches for capturing comparable and reproducible imaging markers across individuals, as well as the disagreement on the amount of data required to robustly detect intrinsic connectivity networks (ICNs) and diagnostically relevant patterns of rsFNC at the individual subject level. Recently, spatially constrained independent component analysis (scICA) has been proposed as an automated method for extracting ICNs standardized to a chosen network template while still preserving individual variation. Leveraging the scICA methodology, which solves the former challenge of standardized neuroimaging markers, we investigate the latter challenge of identifying a minimally sufficient data length for clinical applications of resting‐state fMRI (rsfMRI). Using a dataset containing rsfMRI scans of individuals with schizophrenia and controls (*M* = 310) as well as simulated rsfMRI, we evaluated the robustness of ICN and rsFNC estimates at both the subject‐ and group‐level, as well as the performance of diagnostic classification, with respect to the length of the rsfMRI time course. We found individual estimates of ICNs and rsFNC from the full‐length (5 min) reference time course were sufficiently approximated with just 3–3.5 min of data (*r* = 0.85, 0.88, respectively), and significant differences in group‐average rsFNC could be sufficiently approximated with even less data, just 2 min (*r* = 0.86). These results from the shorter clinical data were largely consistent with the results from validation experiments using longer time series from both simulated (30 min) and real‐world (14 min) datasets, in which estimates of subject‐level FNC were reliably estimated with 3–5 min of data. Moreover, in the real‐world data we found rsFNC and ICN estimates generated across the full range of data lengths (0.5–14 min) more reliably matched those generated from the first 5 min of scan time than those generated from the last 5 min, suggesting increased influence of “late scan” noise factors such as fatigue or drowsiness may limit the reliability of FNC from data collected after 10+ min of scan time, further supporting the notion of shorter scans. Lastly, a diagnostic classification model trained on just 2 min of data retained 97%–98% classification accuracy relative to that of the full‐length reference model. Our results suggest that, when decomposed with scICA, rsfMRI scans of just 2–5 min show good clinical utility without significant loss of individual FNC information of longer scan lengths.

## Graphical abstract

Here, we test the reliability (both in general and in the context of identifying schizophrenia) of intrinsic connectivity networks and functional connectivity patterns estimated via spatially constrained ICA with respect to data length for short (5 min), medium (15 min) and long (30 min) resting‐state fMRI scans. Our results suggest that, when decomposed with spatially constrained ICA, resting‐state fMRI scans of just 2–5 min show good clinical utility without significant loss of individual functional connectivity information or diagnostic accuracy of longer scan lengths.

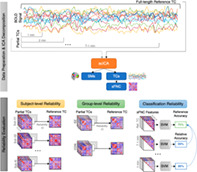

## 1 INTRODUCTION

Resting‐state functional MRI (rsfMRI) has been a valuable tool for identifying and investigating brain networks and their functional interactions, often referred to as resting‐state functional network connectivity (rsFNC), in both typical individuals and those diagnosed with psychiatric and mood disorders. Clinically, rsfMRI offers several benefits, namely that it is non‐invasive, it is relatively easy to administer, and imposes fewer demands on patients than other imaging techniques or task‐based fMRI paradigms, an important consideration for clinical populations that may not be able to perform standardized tasks in the scanner. Studies of rsFNC have also identified characteristic and reproducible connectivity patterns capable of discriminating between various diagnostic groups (Arbabshirani et al., 2013; Li et al., 2020; Liu et al., 2008), as well as “fingerprinting” individuals and predicting behavior (Finn et al., 2015).

While these benefits show promise for rsFNC to serve as a potential biomarker and move towards precision diagnosis in the currently tangled landscape of psychiatric disorders, several factors have prevented widespread clinical adoption of such methods. One such challenge is the lack of standardized approaches for capturing imaging markers, in this case individualized intrinsic connectivity networks (ICNs), that are reproducible and comparable across individuals. Independent components analysis (ICA) is a widely used data‐driven approach for extracting maximally spatially independent components that share co‐varying activation patterns from voxel‐level fMRI data (Calhoun et al., 2001; Durieux & Wilderjans, 2019; Esposito et al., 2005; Gordon et al., 2017; Salehi et al., 2020). Though several group ICA methods have been developed that enforce correspondence between individual‐level ICNs in a given group analysis (Beckmann et al., 2009; Calhoun et al., 2001; Du & Fan, 2013), there is no such guarantee of correspondence across different datasets or studies. To address this challenge, spatially constrained ICA (scICA) methods have recently been proposed (Du et al., 2020) that can extract individualized ICNs guided by the spatial prior of an independently derived and validated network template. The scICA approach is fully automated and ensures the correspondence of ICNs across subjects while maintaining individualized identification of components, suggesting it can be of great use for precision biomarker development.

In addition, there is currently debate in the field surrounding the amount of rsfMRI data needed to generate robust estimates of functional networks and the corresponding resting‐state functional connectivity (rsFC) between them. Typically, rsfMRI scans lengths range from 5 to 15 min, but recent work has yielded conflicting results for “optimal” scan lengths, suggesting as little as 5–6 min (Birn et al., 2013; Braun et al., 2012; Van Dijk et al., 2010) or as much as 30–40 min (Gordon et al., 2017; Milham et al., 2021; Murphy et al., 2007) of data are necessary to produce sufficiently reliable estimates of individual rsFC. While shorter scanning sessions would be more cost‐ and resource‐efficient for clinical implementation, the ICN and rsFC estimates from shorter time courses can possibly be more susceptible to spurious noise; conversely, while longer scanning sessions have the benefit of averaging across more data, the longer a subject spends in the scanner the more susceptible they are to fatigue, increased head motion, drowsiness, and fluctuations in vigilance (Damaraju et al., 2020), which also contribute to noise. Furthermore, individuals with psychiatric or mood disorders can become distressed in the MRI scanner and be unable to tolerate long acquisitions, making the scan duration an increasingly important factor for development of neuroimaging biomarkers with practical clinical utility. Thus, the lack of consensus around an appropriate “minimally sufficient” scan length for clinical applications of rsfMRI has left this an open area of research.

Importantly, existing studies of scan length reliability have focused mainly on atlas‐ and seed‐based approaches. To the best of our knowledge there has been no such examination of reliability using a data‐driven ICA approach, specifically scICA. We suggest that the scan length required to achieve reliable results is at least in part dependent on the methodological approach employed. Thus, a data‐driven method may produce results that differ from seed‐ and atlas‐based approaches. Moreover, we hypothesize that the regularization provided by the spatial priors in scICA serve to stabilize the independent component solution even when less data is used, providing higher reliability at shorter scan lengths than what has been reported for non‐ICA approaches.

Motivated by the lack of consensus in recommended scan lengths for clinical applications, we investigate the robustness of both subject‐specific ICNs extracted via scICA and their resultant rsFNC matrices with respect to time series length. As we are interested specifically in studying minimal sufficient scan lengths in the context of clinical biomarker development, we also evaluate the robustness of scan length to the identification of significant group differences in rsFNC between, as well as classification of, schizophrenia and control groups. To supplement the relatively short clinical scans and study the effect of reference length on minimal sufficient scan lengths, we replicate these experiments both in simulated 30‐min rsfMRI time courses as the reference, as well as in real‐world 14‐min rsfMRI scans of healthy young adults from a subset of the Human Connectome Project.

## 2 MATERIALS AND METHODS

### 2.1 Spatially constrained ICA

**FIGURE 1:**
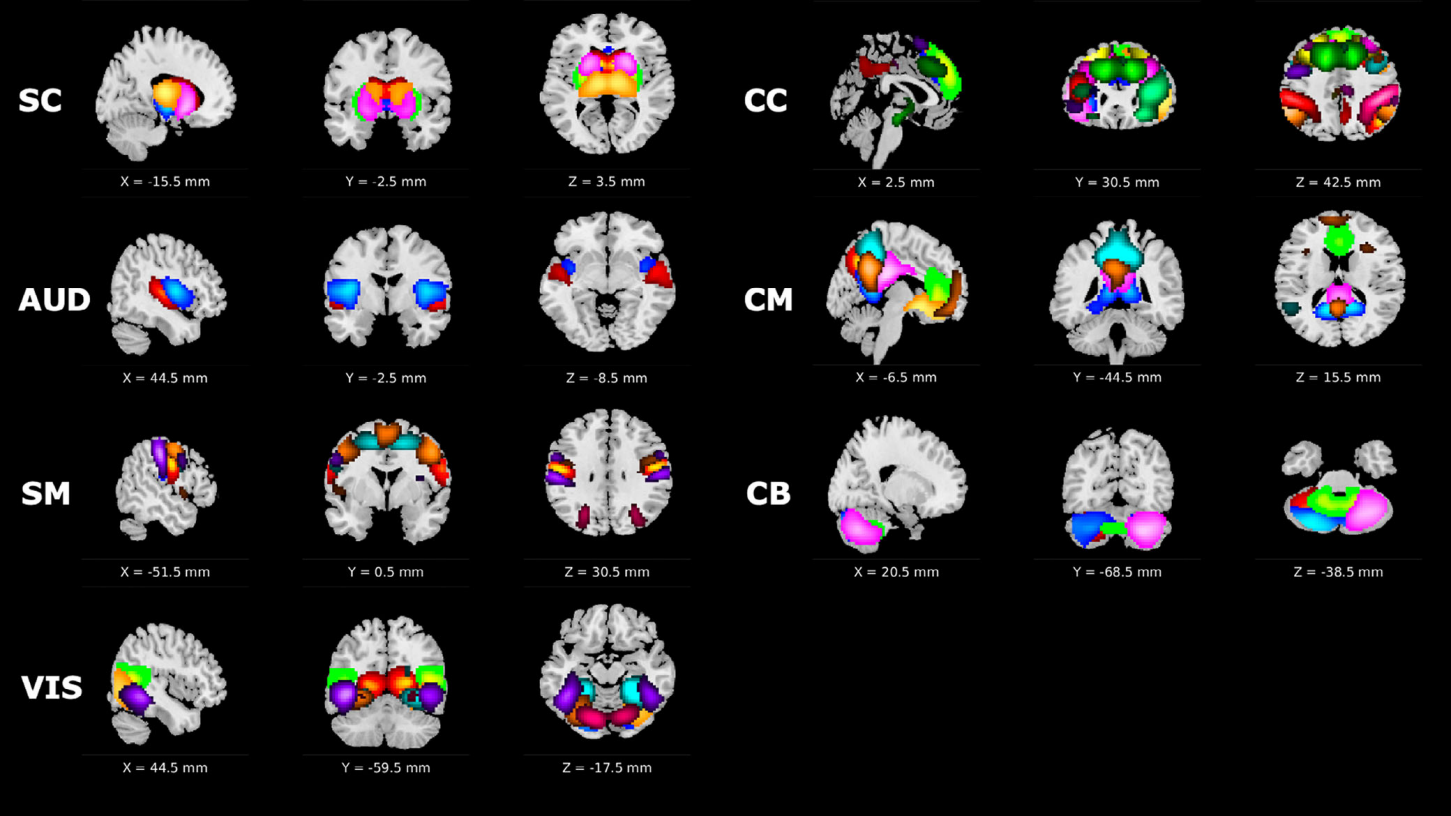
NeuroMark network templates across eight functional domains used for spatially constrained independent component analysis (ICA). Colors represent individual components within each domain. Domain abbreviations: subcortical (SC), auditory (AUD), sensorimotor (SM), visual (VIS), cognitive‐control (CC), default mode (DM), and cerebellar (CB).

A spatially constrained ICA (scICA) approach called multivariate‐objective optimization ICA with reference (MOO‐ICAR) was implemented using the GIFT software toolbox (http://trendscenter.org/software/gift) (Iraji et al., 2021). Briefly, the MOO‐ICAR framework estimates subject‐level independent components (ICs) using existing network templates as spatial guides (Du et al., 2020). The main advantage of the scICA framework is the guaranteed correspondence between the estimated ICs across subjects, but an added benefit is the ability to customize the network template used as the spatial reference in the ICA decomposition, enabling a highly tailored analysis of disease‐specific networks or a more generalized analysis of canonically accepted functional networks suitable for global use across a range of populations. In this work we focused on the latter approach and utilized the NeuroMark_fMRI_1.0 template (described in detail in Du et al., 2020 and available for download at https://trendscenter.org/data/). This template consists of *N* = 53 high fidelity ICNs that were identified and reliably replicated in two large scale datasets (Figure 1) and have been categorized into seven major functional domains: subcortical (SC), auditory (AUD), sensorimotor (SM), visual (VIS), cognitive‐control (CC), default mode (DM) and cerebellar (CB). The NeuroMark_fMRI_1.0 template is considered a global template and was generated using data from control individuals, but importantly this network template was also validated across six different brain disorders including schizophrenia, autism, Alzheimer's disease, mild cognitive impairment, bipolar disorder, and major depressive disorder. This validation confirmed that the ICNs defined in the NeuroMark_fMRI_1.0 template did indeed capture clinically relevant functional entities, and that the connectivity between these entities was capable of identifying functional patterns characteristic of disease (Du et al., 2020), thus suiting our purposes for this study.

The MOO‐ICAR algorithm used to implement the scICA maximizes two objective functions—one function to optimize the overall independence of the networks, and another to optimize the correspondence of each subject‐specific network to its template (Du et al., 2020). Both objective functions, $J\left(S_{l}^{k}\right)$ and $F\left(S_{l}^{k}\right)$, are listed in the following equation, which summarizes how the *l* ^th^ network can be estimated for the *k* ^th^ subject using the network template $S_{l}$ as guidance:

$$\max\left\{\begin{array}{c} J\left(S_{l}^{k}\right)={\left\{E\left[G\left(S_{l}^{k}\right)\right]-E\left[G\left(v\right)\right]\right\}}^{2}\\ F\left(S_{l}^{k}\right)=E\left[S_{l}S_{l}^{k}\right] \end{array}\right.$$

$$s.t.\left\|w_{l}^{k}\right\|=1$$

In this formulation, $S_{l}^{k}={\left(w_{l}^{k}\right)}^{T}\cdot X^{k}$ represents the estimated *l* ^th^ network of the *k* ^th^ subject, where $X^{k}$ is the whitened fMRI data matrix of the *k* ^th^ subject and $w_{l}^{k}$ is the unmixing column vector, to be solved in the optimization functions. The function $J\left(S_{l}^{k}\right)$ serves to optimize the independence of $S_{l}^{k}$ via negentropy. Here, v is a Gaussian variable with mean zero and unit variance, $G\left(\right)$ is a nonquadratic function, and $E\left[\right]$ denotes the expectation of the variable. The function $F\left(S_{l}^{k}\right)$ serves to optimize the correspondence between the template network ($S_{l}$) and subject network ($S_{l}^{k}$). The optimization problem is solved by applying a linear weighted sum to combine the two objective functions, with weights set at 0.5. Applying scICA via MOO‐ICAR to each scan extracts subject‐specific ICNs corresponding to each of the *N* network templates, along with the relevant time courses.

### 2.2 Evaluation framework

**FIGURE 2:**
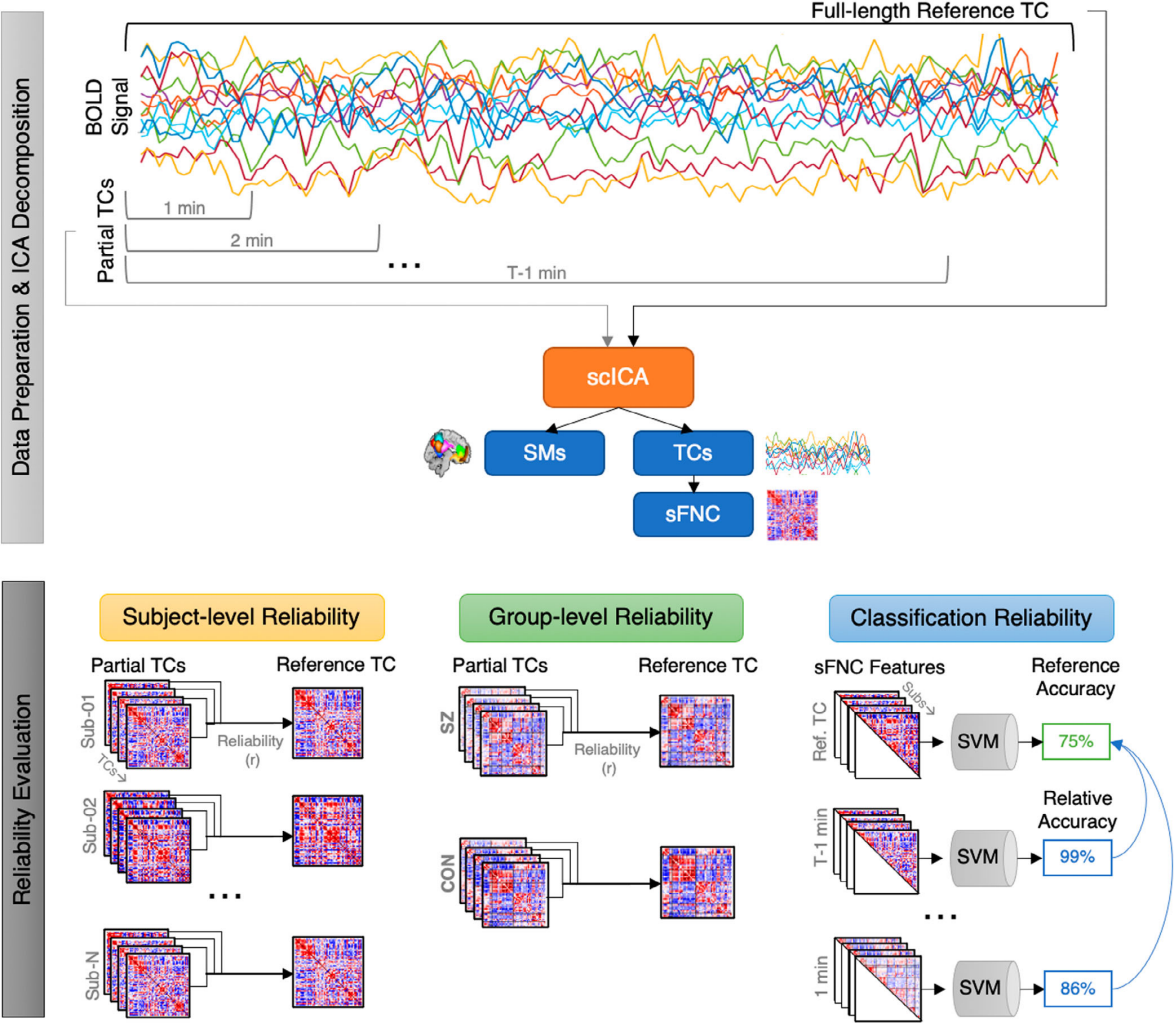
Analysis framework. Data preparation steps including extraction of partial time courses, as well as spatially constrained ICA and its outputs are depicted on the top panel. Reliability evaluation steps including subject‐level, group‐level and classification reliability experiments are depicted on the bottom panel. Abbreviations: time course (TC); blood oxygen level‐dependent (BOLD); independent components analysis (ICA); spatially constrained ICA (scICA); spatial maps (SMs); static functional network connectivity (sFNC); schizophrenia (SZ); control (CON); support vector machine (SVM).

A flowchart of our analysis framework is presented in Figure 2. We began by partitioning the preprocessed rsfMRI data into incrementally longer segments, beginning with the first 1 min, then 2 min, and so on until the full length of the data was reached (due to the increased temporal resolution in the HCP data, the rsfMRI time series were parsed in 30 s increments). Next, we applied scICA via the MOO‐ICAR framework (Section 2.1) separately to each length of rsfMRI, extracting subject‐specific ICNs and their corresponding TCs. Results from the full‐length TC were considered the reference or “gold standard” in our evaluation, thus the robustness of shorter data lengths was evaluated with respect to the full‐length TC. To evaluate the spatial map stability of the scICA decomposition, as well as the rsFNC stability of their respective TCs, we computed subject‐level Pearson correlations between full TC and partial TC experiments for both the spatial composition of the extracted ICNs and the resultant rsFNC matrices. We utilized a robustness threshold of correlation ≥0.85 to the reference (i.e., full‐length TC), formerly proposed in Gordon et al. (2017), to identify a “minimally sufficient” data length with respect to these metrics. Pearson's r was used to assess reliability rather than intraclass correlation (ICC) to enable better comparison to, and use of the same evaluation criteria of, this prior work.

In each experiment, subject‐level static functional network connectivity (sFNC) was computed via pairwise Pearson correlation between time courses of all ICNs, resulting in an *N* × *N* sFNC matrix. Fisher's *Z* transform was applied to all sFNC matrices to improve normality. We computed group‐average sFNC matrices for the schizophrenia (SZ) and control (CON) groups (or groups A and B in the simulated data) at each data length. We investigated group differences in sFNC values between the SZ and CON (or Group A/B) populations using two parallel approaches—two‐sample *t*‐test as well as univariate multiple linear regression including age, gender, scanning site and head motion as covariates. Significant group differences in sFNC were identified as relationships whose *p*‐values survive FDR correction at *α*_FDR_ = 0.05. To further evaluate the stability of diagnostically relevant rsFNC patterns as the length of fMRI data decreased, we computed group‐level correlations between the full TC and each of the partial TCs for each of the mean SZ/CON sFNC matrices, *t*‐test *t*‐values, and diagnosis term t‐values from the multiple linear regressions.

We apply this evaluation framework to our discovery rsfMRI dataset (*M* = 310 subjects) to examine scan length reliability as it relates to SZ/CON group differences, as well as to our simulated dataset (*M* = 100 subjects) and the HCP dataset (*M* = 98 subjects, 392 scans) to examine scan length reliability in longer time series (Section 2.4).

### 2.3 Group classification

We further investigate the robustness and clinical utility of scICA‐based estimates of rsFNC with a group classification task. Using our discovery rsfMRI dataset as training data (*M* = 310 subjects; 150 SZ), we generated subject‐level feature vectors for each data length by extracting the upper triangular of the corresponding scICA‐derived sFNC matrix. Using the “fitclinear” function in MATLAB, we fit binary LASSO‐regularized linear SVM classification models separately for each data length (1, 2, 3, 4, and 5 min) to classify each subject as SZ/CON. For each model, the lambda parameter was tuned using five‐fold cross validation. After obtaining the optimal lambda value, performance for each of the five models was estimated with 500 rounds of bootstrap resampled five‐fold cross validation. The final five models were trained on the full training dataset and tested on the held‐out independent validation dataset (*M* = 129 subjects; 50 SZ) using the same bootstrap resampling scheme (500 rounds) for external evaluation of classification performance and generalizability at each data length.

### 2.4 Data and preprocessing

#### 2.4.1 Clinical fMRI data

We utilized datasets from two existing projects in this study: the FBIRN (Functional Imaging Biomedical Informatics Research Network) dataset was used as our discovery dataset, and the COBRE (Center for Biomedical Research Excellence) dataset was used as a validation dataset in our classification experiments. We selected a subset of subjects from each dataset that satisfy the following inclusion criteria: (a) data of individuals with typical control or schizophrenia diagnosis; (b) data with high‐quality registration to echo‐planar imaging (EPI) template; and (c) head motion transition of less than 3° rotation and 3‐mm translation in every direction (Fu et al., 2021). This data selection resulted in an age‐ and gender‐matched discovery dataset including 150 individuals with schizophrenia (SZ) and 160 controls (CON) (Keator et al., 2016). FBIRN resting‐state fMRI (rsfMRI) data were collected with 3‐T MRI scanners with a repetition time (TR) of 2 s, voxel size of 3.44 × 3.44 × 4 mm, a slice gap of 1 mm, and a total of 157 volumes. Subjects were instructed to keep their eyes closed during the resting‐state scan but not fall asleep. Additionally, the validation dataset consisted of 50 SZ and 79 CON samples (Aine et al., 2017). The COBRE rsfMRI data were collected with 3‐T MRI scanners with a TR of 2 s, voxel size of 3.75 × 3.75 × 4.55 mm, and a total of 145 volumes. In the validation set, subjects were instructed to keep their eyes open and passively stare at a fixation cross. Details on age and sex demographics of subjects in both datasets are listed in Table 1. Informed consent was obtained from each participant prior to scanning and all studies were approved by the Institutional Review Boards of the corresponding institutions (University of California: Irvine, San Diego, Los Angeles; Stanford University, University of New Mexico, University of Iowa, University of Minnesota, Duke University, University of North Carolina, Brigham and Women's Hospital, Massachusetts General Hospital, Yale University). For both data sets, preprocessing included brain extraction, slice‐timing, and motion correction steps. Preprocessed data were then registered into structural MNI space, resampled to 3 mm^3^ isotropic voxels, and spatially smoothed using a Gaussian kernel with a 6 mm full‐width at half‐maximum (FWHM) on a per‐subject basis. Finally, the first five timepoints were trimmed from the time course and all voxel time courses were *z*‐scored.

**TABLE 1:** Subject demographic information.

Dataset	Diagnostic group	*N*	Sex	*N*	Age (years)	
					Mean ± SD	Median (range)
FBIRN (discovery)	CON	160	Male	115	37.26 ± 10.71	39 (19–59)
			Female	45	36.47 ± 11.33	33 (19–58)
	SZ	150	Male	114	38.74 ± 11.78	40 (18–62)
			Female	36	39.06 ± 11.40	36 (21–57)
COBRE (validation)	CON	79	Male	55	39.07 ± 12.43	38 (18–65)
			Female	24	34.92 ± 10.23	34 (18–58)
	SZ	50	Male	42	37.43 ± 15.05	32.5 (19–64)
			Female	8	43.25 ± 12.78	40 (31–65)

Abbreviations: COBRE: Center for Biomedical Research Excellence; CON: control; FBIRN: Functional Imaging Biomedical Informatics Research Network; SZ: schizophrenia.

#### 2.4.2 Simulated data

**FIGURE 3:**
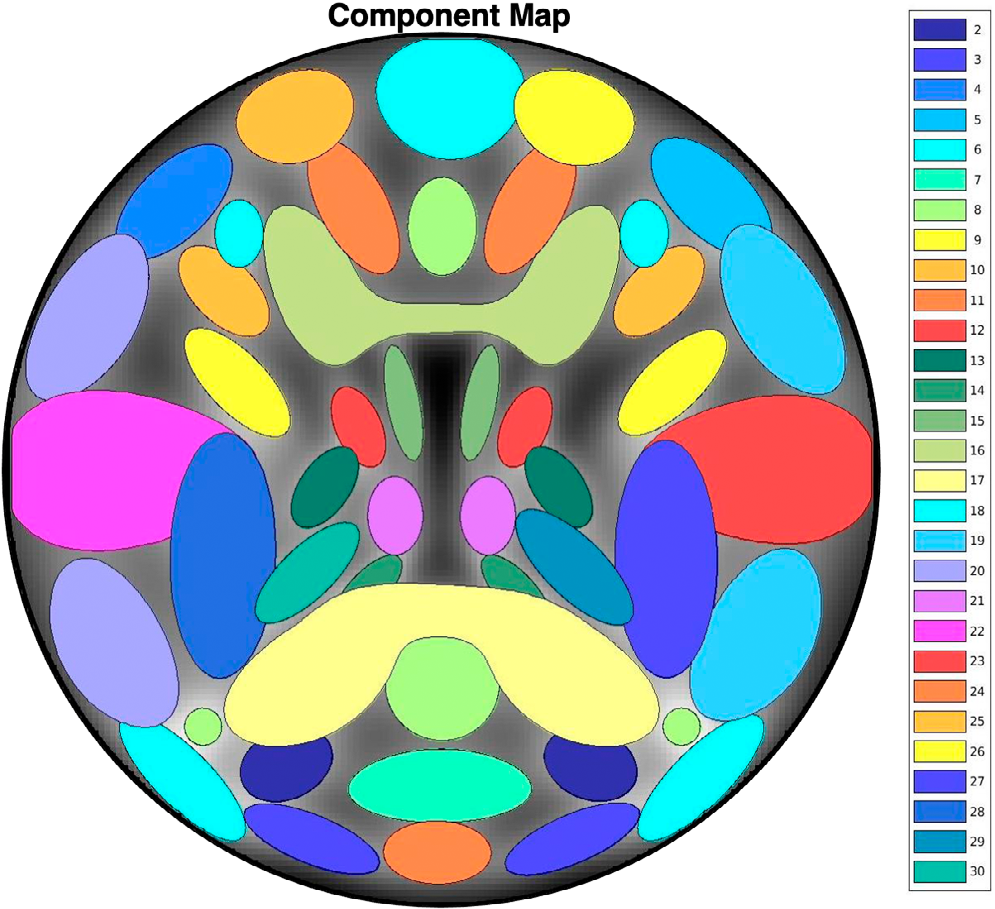
Spatial map of the 29 components used in our simulation study (corresponding to default components numbers 2–30 in the SimTB software).

To supplement the relatively short data lengths available from our clinical rsfMRI datasets, we simulated a set of longer fMRI time courses using the SimTB toolbox (Erhardt et al., 2012) (freely available for download at https://trendscenter.org/software/simtb/). Briefly, SimTB utilizes a data generation model under the assumption of spatiotemporal separability, meaning the simulated fMRI data can be expressed as the product of time courses (TCs) and spatial maps (SMs). These simulated data are modeled with realistic dimensions, spatiotemporal activations, and noise characteristics typical of fMRI datasets (Allen et al., 2012). For subjects *i* = 1, …, *M*, we model *C* components, each consisting of a SM and corresponding TC. In our simulation, we set *M* = 100 subjects and *C* = 29 components. SMs have *V* = 148 × 148 voxels and TCs are *T* = 900 time points in length with a TR = 2 s (totaling 30 min of data). The *C* = 29 components used in our simulation are shown in Figure 3. In our simulation, individual variability in component SMs is implemented as follows. SMs are translated according to a bivariate normal distribution with mean zero and standard deviation of 0.75 voxels and are rotated by normally distributed angles with mean zero and a standard deviation of 1°. SM size is also randomly varied, with the spread parameter, *ρ*, uniformly distributed between 0.85 and 1.15. Additionally, component amplitudes, *g*_*ic*_, are distributed normally with a mean of 3 and standard deviation of 0.3, simulating individual TC variation.

We model two groups, A and B, that differ in four ways, modeled after a similar experiment in (Allen et al., 2012) and described in detail below. Each group has 50 subjects, for *M* = 100 subjects total. Group differences are as follows. (1) Group A has larger amplitude for component 7 than Group B. This is modeled by distributing *g*_*i*7_ as normal with mean 3.5 and standard deviation 0.3 for Group A, and mean 2.5, standard deviation 0.3 for Group B. (2) Groups A and B have different shapes for a network composed of components 5 and 10. The network is modeled by assigning shared events between components 5 and 10 and setting the amplitude of unique events, *A*_u_ = 0, creating identical TCs. For Group A, the amplitude of component 5 is *g*_*i*5_ = 0.7 × *g*_*i*10_, whereas for Group B *g*_*i*5_ = 1 × *g*_*i*10_. Thus, Group A has a network where the component 5 node is weaker than the component 10 node and Group B has two nodes of equivalent strength. (3) Groups A and B have different shapes and different amplitudes for a network composed of components 22 and 23. For Group A, *g*_*i*23_ = 0.7 × *g*_*i*22_, where *g*_*i*22_ is distributed normally with mean 6 and standard deviation 0.3. For Group B, *g*_*i*23_ = 1 × *g*_*i*22_, where *g*_*i*22_ is distributed normally with mean 3 and standard deviation 0.3. Thus, Group A has a lateralized network where the left node is stronger than the right and Group B has a bilaterally symmetric network. Furthermore, the amplitude of the network for Group A is much larger than the amplitude for Group B. (4) Group B has stronger FNC between components 3 and 4 than Group A. This is modeled by first designating shared events between components 3 and 4, then distributing *A*_u_ as uniform between [0.5, 1.0] for Group A and between [0.65, 1.15] for Group B.

#### 2.4.3 HCP data

Finally, we applied our evaluation framework on data from a subset of *M* = 100 subjects from the Human Connectome Project (HCP) S1200 dataset (Van Essen et al., 2013). We utilized the HCP data as an additional real‐world evaluation set for our reliability analyses, capitalizing on the relatively long scan length (~15 min) of the rsfMRI acquisitions. However, the “healthy control” nature of the HCP cohort precluded the possibility of group analyses, therefore we conducted the HCP experiments for the purpose of supplemental validation of subject‐level scan length reliability. Each rsfMRI scan in the HCP dataset consists of 1200 time points at a TR = 0.72 sec and a voxel size of 2 mm isotropic. Preprocessing of HCP data was identical to that of the FBIRN and COBRE data sets described in Section 2.4.1. The HCP data consisted of four rsfMRI scans per subject, two collected on the initial visit (labeled REST1_LR and REST1_RL) and two on a follow up visit (REST2_LR and REST2_RL). Two subjects from our initial subset did not have complete data for all four scans and were excluded from analysis, resulting in a final sample size of *M* = 98 subjects and 392 rsfMRI scans in total.

The inherent differences in spatial and temporal resolution in the acquisitions of the FBIRN and HCP datasets have been shown to result in considerable differences in spatial smoothness of ICN estimations, namely resulting in smoother spatial maps in the FBIRN data (Iraji et al., 2022). To mitigate the effects of these smoothness differences on our reliability estimates, we applied post‐hoc spatial smoothing to the HCP data at several levels (range FWHM = 3–10). We computed a smoothness score, defined as 1—normalized average gradient across the spatial maps of all 53 ICNs, at each level of smoothing as well as for the original unsmoothed HCP and FBIRN datasets. Finally, we identified the level of post‐hoc smoothing in the HCP data that most closely matched the smoothness of the FBIRN data for the same scan time (FWHM = 3.53) (Figure S1), which was used for further reliability analyses.

## 3 RESULTS

### 3.1 Subject‐level estimates of ICNs and sFNC are highly robust to data length

**FIGURE 4:**
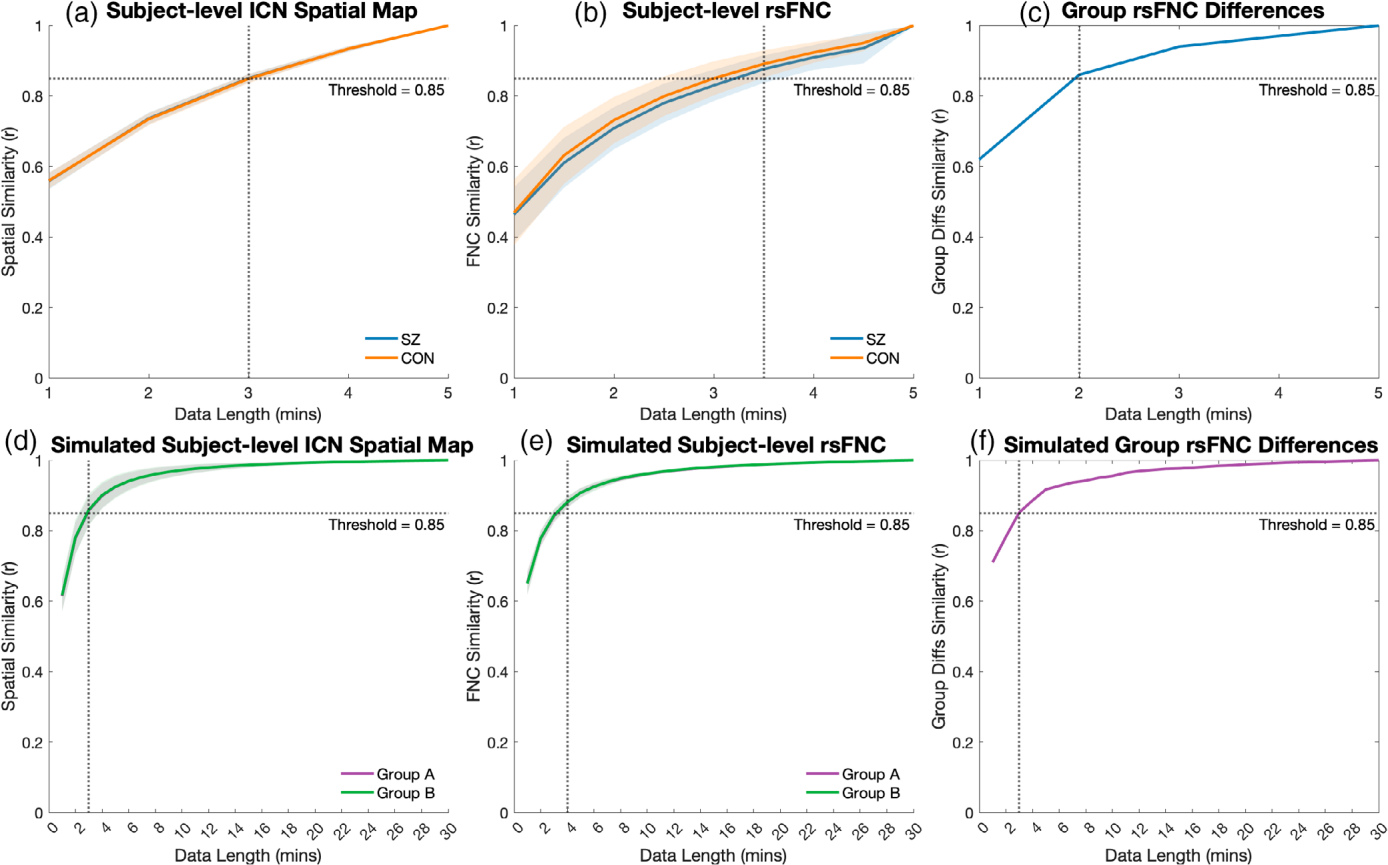
Reliability results for clinical (a–c) and simulated (d–f) datasets. Subject‐level measures (a and b, d and e) show mean (solid line) and standard deviation (shaded area) across all subjects. Dotted lines indicate data lengths at which the measures meet or exceed the robustness threshold (*r* ≥ 0.85; Gordon et al., 2017). CON, control; ICN, intrinsic connectivity network; rsFNC, resting‐state functional network connectivity; SZ, schizophrenia.

We evaluated the stability of subject‐level estimates of ICN spatial maps and sFNC matrices derived via scICA with respect to the length of rsfMRI data (Figure 4a,b). We observed increasing variation in the stability of subject‐level sFNC estimates at shorter data lengths, but comparatively little variation in the ICN spatial stability, likely owing to the regularization provided by the spatial priors in the scICA decomposition. Similarly, we found no discernible group differences between SZ and CON in ICN spatial map stability, whereas the CON group exhibited slightly higher stability in sFNC than the SZ group at shorter data lengths. Results showed only 3 min of rsfMRI data were sufficient to meet the robustness threshold for the subject‐specific ICN spatial maps and 3.5 min were sufficient for the corresponding subject‐level sFNC estimates.

### 3.2 Group‐level sFNC patterns can be reliably estimated with less data

**FIGURE 5:**
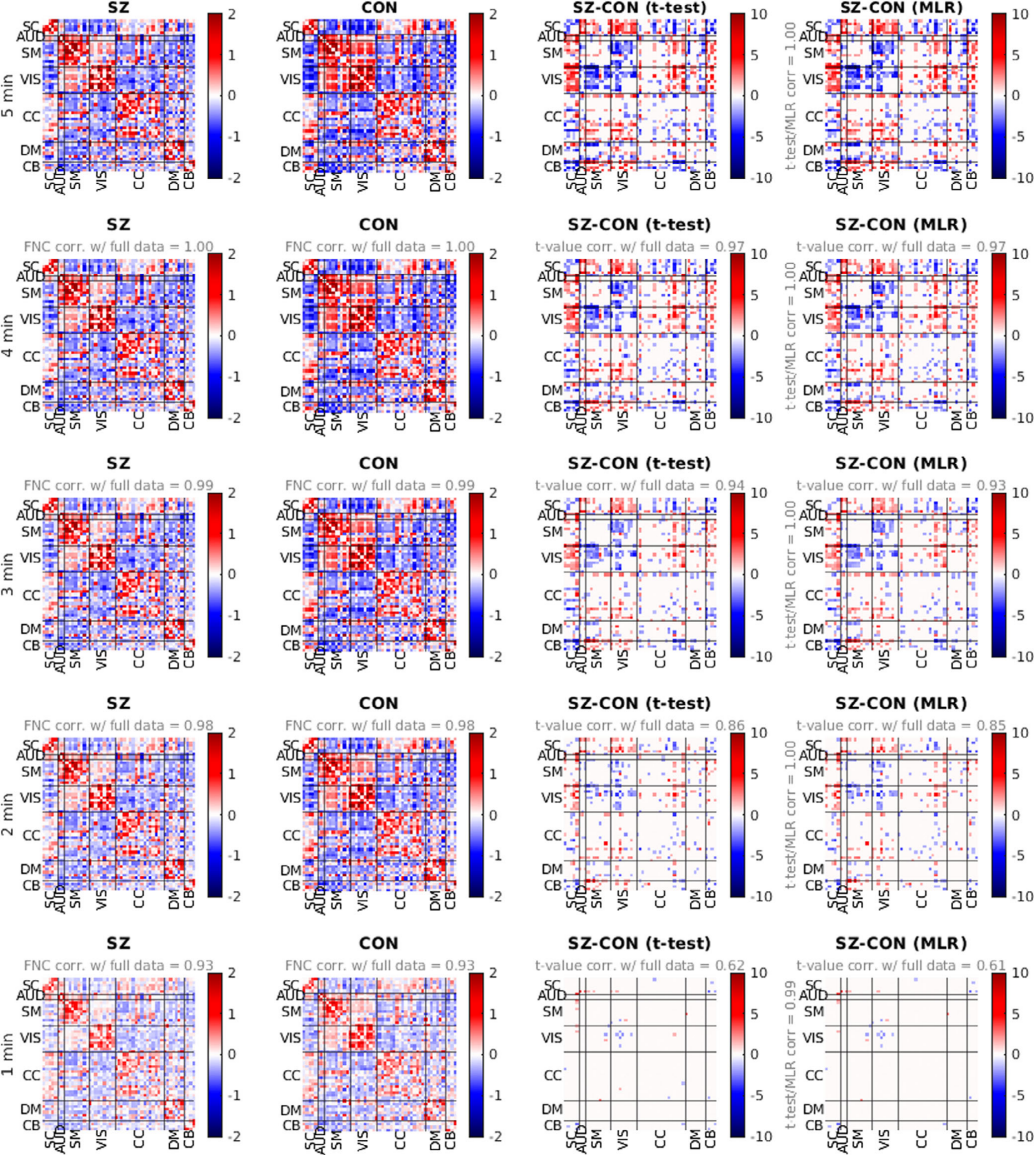
(a and b) Mean functional network connectivity (FNC) for patients with schizophrenia (SZ) (a) and typical controls (CON) (b) from the full (5 min) and partial (1–4 min) fMRI time course. For experiments on partial time series data, Pearson correlation with FNC from the full data is reported. (c and d) Group differences (SZ‐CON) in FNC. Values are plotted as −log10(*p*‐value) × sign(*t*‐value), where statistics are obtained via t‐test across diagnosis groups (c) or from the diagnosis term in univariate multiple regression (MLR) models (d). For experiments using partial time series data, *t*‐value correlation with full data experiments is reported.

In addition to subject‐level reliability of ICN and sFNC estimates, we evaluated the reliability of group‐level sFNC patterns across TC lengths. Results showed the characteristic rsFNC signatures for the SZ and CON groups were highly robust to the length of rsfMRI data used (Figure 5a,b), indicating high group‐level rsFNC stability. The group‐level sFNC matrices derived from even 1 min of data were highly correlated to the full TC reference for both the SZ (*r* = 0.94) and CON (*r* = 0.93) groups, and this relationship continually increased as more of the rsfMRI time course was utilized.

We found significant group differences in sFNC patterns across all experiments, both with the classic t‐test and with multiple regression with added covariates (Figure 5c,d). Results showed near‐perfect concordance between these two parallel methodologies, with correlations in t‐values ≥0.99 in each experiment. We found only 2 min of rsfMRI data were required to meet the robustness threshold (correlation ≥0.85 to the reference) and reliably estimate the significant group differences identified from the full TC (Figure 4c). Specifically, the results showed that the group differences that were most robust to data length were lower within‐domain connectivity of the VIS domain and higher cross‐domain connectivity between SC‐SM and SC‐VIS domains in the SZ group compared to that of the CON group.

### 3.3 Simulation study suggests ICN and sFNC estimates from relatively long rsfMRI can be reliably estimated from very short TCs

**FIGURE 6:**
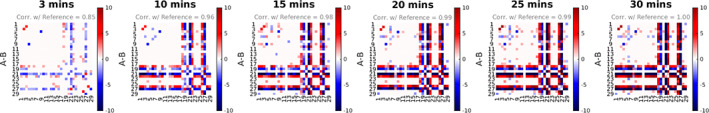
Group differences in rsFNC between Groups A and B in the simulated dataset across various data lengths. Values are plotted as −log10(*p*‐value) × sign(*t*‐value).

To evaluate how reliability is affected with longer rsfMRI as the reference, we simulated 30 min of rsfMRI data designed to model two groups with distinct activation and FNC patterns (Section 2.4.2). The stability of subject‐level estimates of ICN spatial maps and sFNC matrices derived via scICA with respect to the length of the simulated rsfMRI data is shown in Figure 4d,e. Somewhat contrary to results of the SZ/CON data, we observed slightly larger variation in subject‐level spatial map stability at shorter data lengths compared to that of subject‐level sFNC. This could be due in part to the induced variability in translation/rotation/size of the simulated ICN spatial maps. The results showed that the robustness threshold of mean correlation ≥0.85 to the reference was reached with 3 min of data for the spatial composition of subject‐specific ICNs and 4 min of data for subject‐level sFNC, corresponding well to the results from the clinical data and suggesting individualized rsFNC features averaged across even relatively long TCs can be reliably estimated from just a fraction of the time series. Group‐level mean sFNC was highly robust to data length, and similarly we found 3 min of data were required to reach the robustness threshold for significant group differences in sFNC (Figures 4f and 6).

### 3.4 Scan length reliability of ICN and FNC estimation validated in real‐world HCP data

**FIGURE 7:**
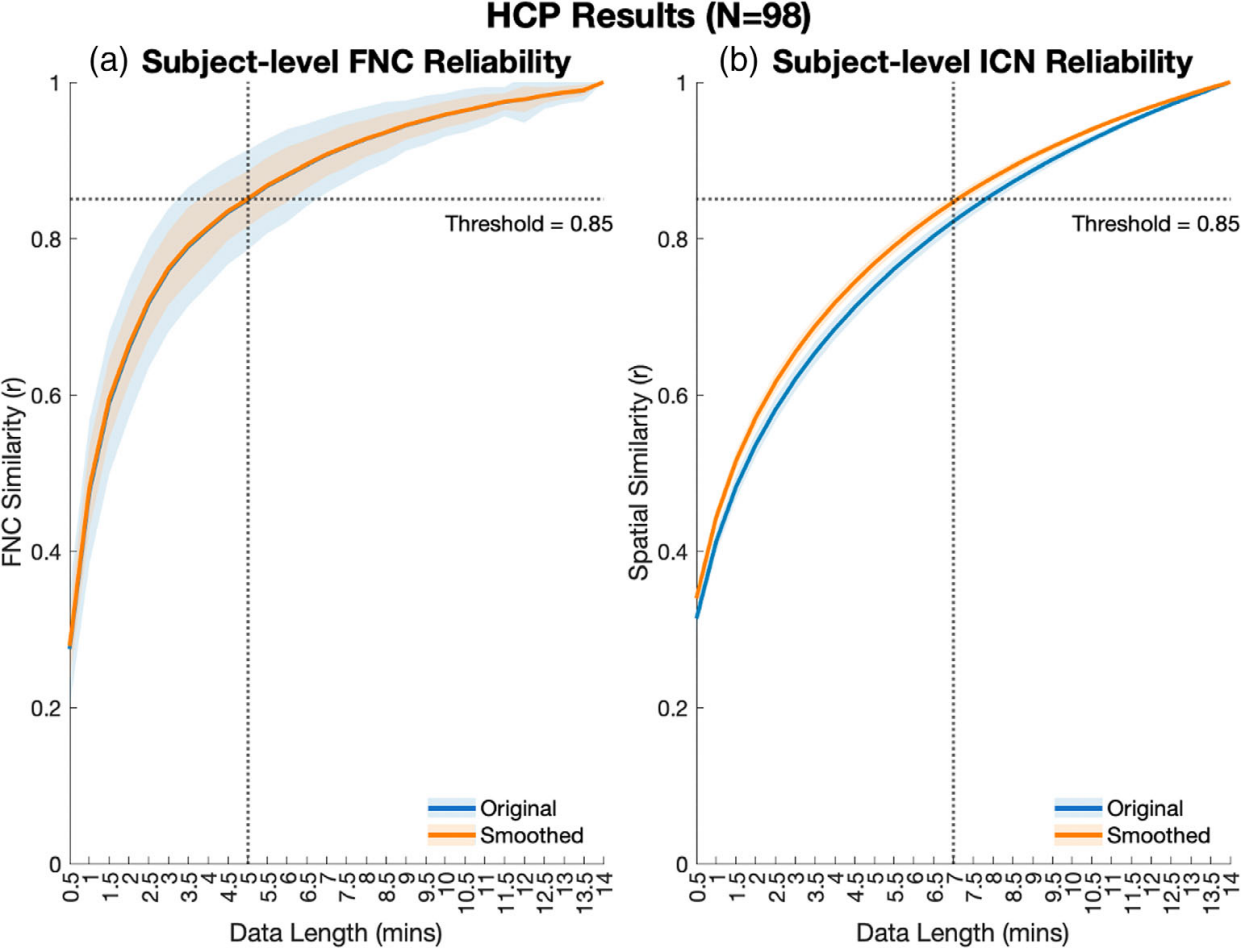
Reliability of FNC (a) and ICN (b) for both original and smoothed (FWHM = 3.53 mm) HCP data. Means are computed across all subjects and sessions (*n* = 392 scans).

As an additional validation in real‐world rsfMRI with relatively longer scan times, we performed reliability analysis on a total of 392 scans from a subset of 98 subjects in the HCP. Results showed that FNC estimates from 5 min of data and ICN spatial map estimates from 7 min of data were sufficient to reliably estimate the FNC and ICN spatial maps from the full‐length time series, here 14 min (Figure 7). Comparison of the original and smoothed datasets showed that smoothing did slightly improve the reliability of subject‐level ICN estimation and did not affect the mean FNC reliability, though it did decrease the standard deviation of the FNC reliability scores compared to the original data. These results suggest the information captured within the 14‐min HCP scans could be reliably estimated even with a 50%–64% decrease in scan length

**FIGURE 8:**
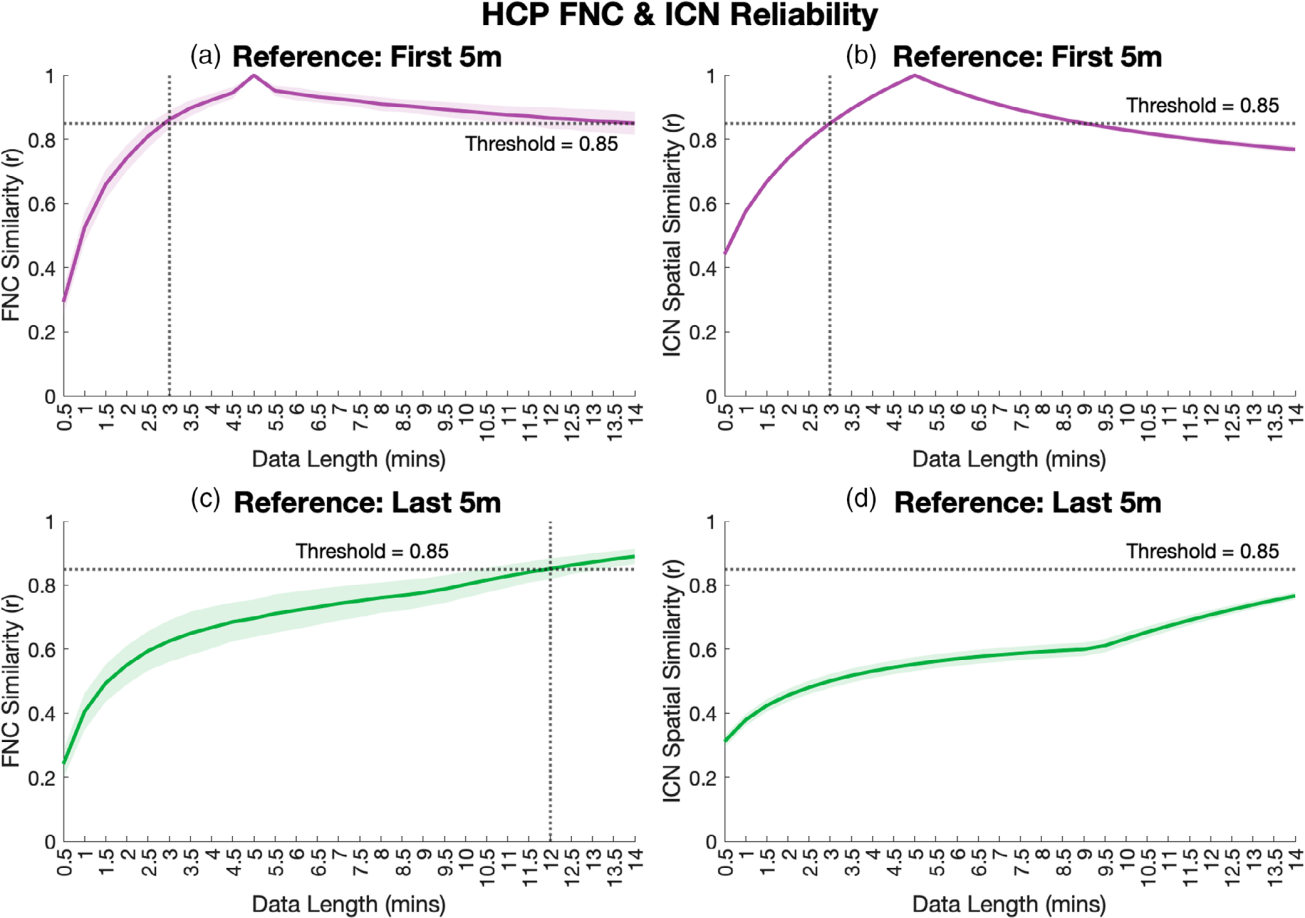
Reliability results using the first 5 min (top row) and last 5 min (bottom row) of data as reference.

The selection of the “gold standard” reference in reliability analyses is critical, as results can vary widely depending on the choice of reference. One issue with longer scan lengths is the susceptibility of fatigue, increased head motion, and drowsiness to pollute the signal, especially at the tail end of the scan. Thus, the use of longer scans that may be affected by these “late scan” noise factors as references could artificially deflate reliability estimates compared to studies that use shorter scans as reference. To evaluate the effects of both the reference length and end‐of‐scan drift, we repeated our reliability analyses using just the first 5 min as well as the last 5 min as reference (Figure 8). When the first 5 min of data were used as reference the results show just 3 min of data were sufficient to reliably estimate subject‐level ICN spatial maps and FNC, concordant with the 5‐min FBIRN results (Section 3.1). In the case of FNC, the reliability remained above the threshold across the full‐length of the 14‐min time series, while ICN spatial map reliability fell below the threshold at 9.5 min or more. Conversely, when the last 5 min of data were used as reference, FNC reliability was not reached without a minimum of 12 min of data and ICN spatial map estimates did not reach reliability with any portion of the time series. Furthermore, we found poor concordance between FNC (Figure 8c) and ICN spatial map (Figure 8d) estimates from the first 5 min and the last 5 min of data (*r* = 0.69, 0.55, respectively), suggesting a large discrepancy in the functional information captured between early‐ and late‐scan rsfMRI. It is possible that these discrepancies could be attributed to brain dynamics, indicating for example a transition between functional states. In fact, such functional dynamics are likely occurring throughout the time series on a subject‐to‐subject basis; however, the linear trend across time for both FNC and ICN reliability is instead more indicative of a stronger global phenomenon across all subjects of the steadily increasing influence of “late scan” noise and fatigue. This trend held when the last 4, 3, 2, and 1 min of the scan were used as reference as well, further supporting this premise (Figure S2).

Overall, the results of our HCP analyses suggest that the information collected within relatively long and temporally granular rsfMRI time series can be reliably estimated from just the first ~20%–50% (3–7 min) of data. Furthermore, these results indicated decreased reliability of functional estimates generated from late‐scan data across the full range of data lengths (0.5–14 min) compared with early‐scan data, likely due to increasing noise as a function of scan time and further supporting the notion of minimized scan times.

### 3.5 Classification accuracy remained stable as less rsfMRI data used for prediction

**FIGURE 9:**
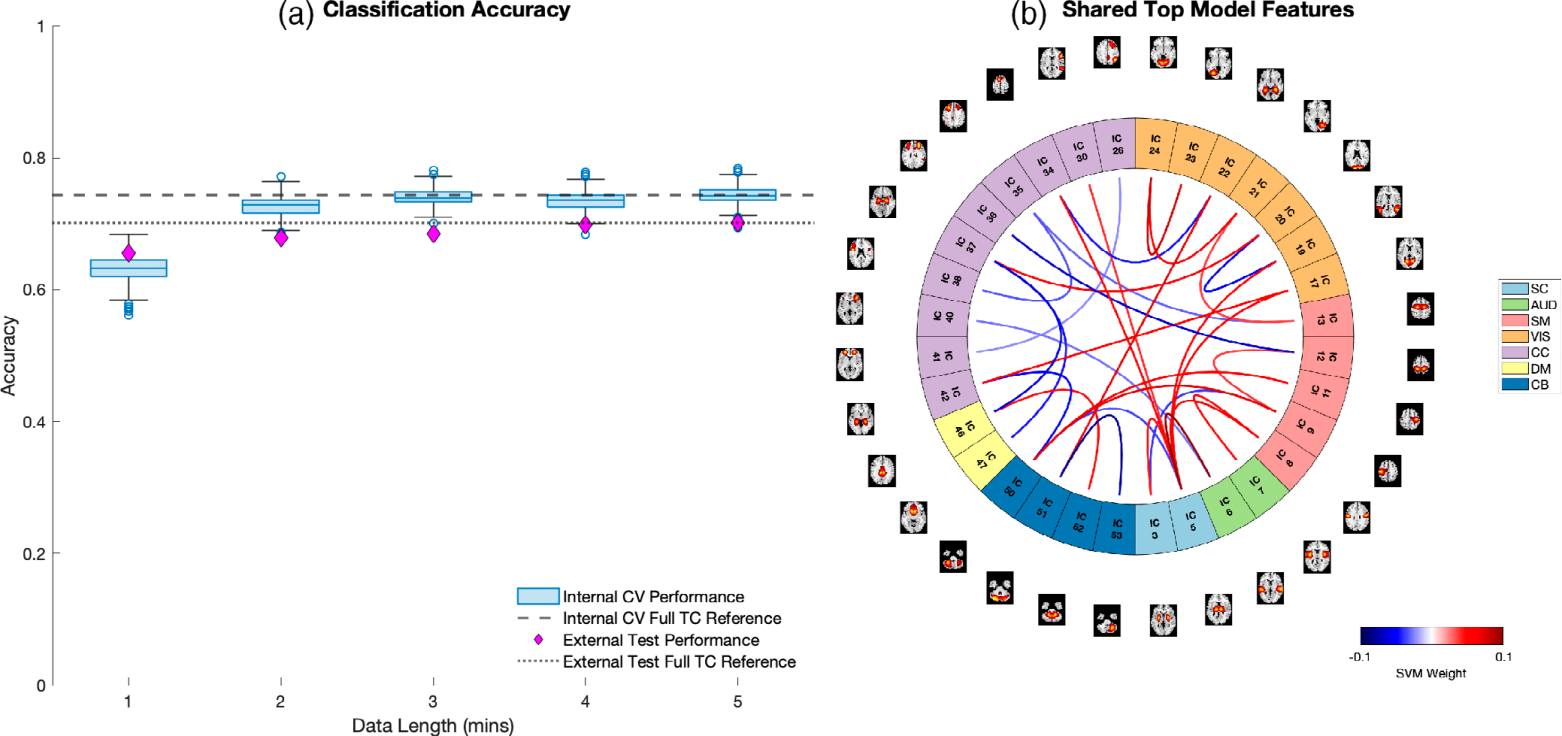
(a) Classification performance of linear SVM models trained at each data length. (b) FNC edges in the top 20% of SVM feature weights shared among all five models.

The results from our classification experiments showed highly stable classification accuracy for models fit across the range of 2–5 min, with the model trained on 2 min of data attaining a relative performance to that of the full TC reference model of 98% in the internal cross validation (0.73 vs. 0.74) and 97% in the external validation (0.68 vs. 0.70) (Figure 9a). A full report of mean classification performance metrics including accuracy, AUC, sensitivity and specificity is listed in Table 2. Notably, the results showed that 4 min of data had a relative performance of 97%–100% compared to the reference TC across all four classification metrics, followed by 95%–100% for 3 min of data, 94%–100% for 2 min of data, and 79%–90% for just 1 min of data. These results suggest that in addition to being robust on an individual level, sFNC features derived from scICA decomposition of rsfMRI data capture clinically relevant patterns of rsFNC, even from very short scan lengths, and yield diagnostic classifications that are highly robust to data length.

**TABLE 2:** Classification results.

	Internal five‐fold CV results (500× bootstrap)								External validation results (500× bootstrap)							
	Accuracy		AUC		Sensitivity		Specificity		Accuracy		AUC		Sensitivity		Specificity	
	Obs.	Rel.	Obs.	Rel.	Obs.	Rel.	Obs.	Rel.	Obs.	Rel.	Obs.	Rel.	Obs.	Rel.	Obs.	Rel.
5 min	0.74	‐	0.83	‐	0.71	‐	0.78	‐	0.70	‐	0.78	‐	0.70	‐	0.70	‐
4 min	0.73	*0.99*	0.82	*0.98*	0.69	*0.97*	0.78	*1.00*	0.70	*0.99*	0.78	*1.00*	0.69	*0.99*	0.71	*1.00*
3 min	0.74	*1.00*	0.81	*0.97*	0.70	*0.99*	0.78	*1.00*	0.68	*0.98*	0.76	*0.97*	0.67	*0.95*	0.71	*1.00*
2 min	0.73	*0.98*	0.80	*0.97*	0.67	*0.95*	0.78	*1.00*	0.68	*0.97*	0.74	*0.94*	0.67	*0.96*	0.68	*0.97*
1 min	0.63	*0.85*	0.69	*0.83*	0.56	*0.80*	0.70	*0.90*	0.57	*0.82*	0.65	*0.82*	0.56	*0.79*	0.60	*0.85*

*Note*: The relative accuracy is reporting the accuracy of the reduced data models to that of the full data model, i.e. the accuracy of the full 5 min model in the CV is 0.74 and the accuracy of the reduced 4 min model in the CV is 0.73, thus the relative accuracy of the 4 min model is 0.73/0.74 = 0.99. We italicized these values to provide a clear visual distinction between observed and relative metrics.

Report of the mean classification accuracy, area under the receiver operating characteristic curve (AUC), sensitivity and specificity for both internal cross validation (CV) and external testing experiments for SVM models trained on features derived from all data lengths (1–5 min). We report both the observed (Obs.) values as well as the relative (Rel.) values compared to that of the full‐length reference time course (5 min).

For each of the five models we also extracted the most influential sFNC edges, defined as the top 20% of features with the highest magnitude weights. We found 31 sFNC edges were commonly included among the top feature weights across all five models (Figure 9b), which mainly belonged to the subcortical (SC), visual (VIS) and sensorimotor (SM) domains. These results align with the significant results of our group differences analysis and involve domains that have previously been implicated in relation to schizophrenia in the literature (Damaraju et al., 2014; Ma et al., 2020).

## 4 DISCUSSION

There is increasing agreement within the psychiatric field that current behavioral diagnostic criteria are likely insufficient to properly disentangle the complex and blurred boundaries between psychiatric disorders as they are presently defined. In light of this, much research is focused on the identification and development of biomarkers for psychiatric disorders, with considerable attention on neuroimaging‐based methods, such as rsfMRI (Arbabshirani et al., 2013). Resting‐state fMRI approaches are well suited for clinical biomarkers, not only because of their non‐invasive nature and relatively high spatial and temporal resolution, but also due to the low demand placed on patients during resting‐state acquisitions, which is especially important for use in psychiatric populations that may experience difficulty performing structured cognitive tasks in the MRI scanner. Another key consideration in the development of psychiatric rsfMRI biomarkers is the duration of image acquisition required. Minimizing the time in the scanner is critical, both for maximizing the comfort of patients, many of which cannot tolerate prolonged containment within the MRI scanner, and for resource efficiency of high‐demand MRI equipment. However, lack of consensus for a minimally sufficient rsfMRI scan length has factored into the delayed adoption of neuroimaging biomarkers in clinical settings. Previous reports have produced conflicting results, proposing that as little as 5–6 min (Braun et al., 2012; Van Dijk et al., 2010) and as much as 30–40 min (Gordon et al., 2017; Milham et al., 2021; Murphy et al., 2007) are necessary to produce robust and reliable estimates of individual rsFNC, with most recent works advocating for longer acquisitions (Noble et al., 2019).

Here, we contribute to this debate by providing an evaluation of reliability of both subject‐ and group‐level measures of rsFNC for individuals with schizophrenia and controls with respect to the length of rsfMRI data used for analysis. Our work differs from existing studies mainly in our use of a spatially constrained ICA approach, which leverages existing network templates as spatial priors to guide the spatiotemporal decomposition of the fMRI data. The data‐driven nature of scICA enables individualized identification of ICNs while simultaneously ensuring their correspondence to a validated set of functional networks of interest and automatedly discarding noise components. We hypothesized that the additional regularization provided by the use of network templates in scICA may serve to enable robust and reliable estimation of rsFNC at shorter scan lengths than those previously reported in when utilizing atlas‐ or seed‐based analyses. Beyond regularization, the scICA has several additional benefits that are favorable for use in clinical settings. Firstly, scICA is completely automated, negating the need for any manual annotation of relevant brain regions from noise components. Secondly, scICA provides crucial correspondence in components between subjects, allowing it to directly patch into any downstream steps in the biomarker algorithm without the need for intermediary steps such as computation of spatial overlaps. Thirdly, scICA is fully parallelizable and can be applied to each patient's scans independently without the need for group analysis. Lastly, scICA can be used with any customized spatial templates that contain ICNs of interest, allowing for additional tailoring to specified networks deemed relevant for any given diagnosis. Though, it is worth noting that the NeuroMark template used in this study is a global template appropriate for use across a range of adult populations, and has been validated to capture disease‐specific patterns in several cohorts with psychiatric or neurological conditions (Du et al., 2020).

There are a few key themes that emerged from the results of our reliability analyses. First and foremost, our results show that the same fundamental subject‐level functional information can be estimated from half as much data (or less) when scICA is used. Across the board, we found that less than 10 min, and often just 5 min or less, of rsfMRI data was sufficient to reliably estimate both ICN spatial maps and patterns of FNC generated from longer “full‐length” reference scans ranging from 5 to 15 to 30 min in length. This result is in concordance with other works that found 5–10 min of data resulted in stable estimations of ICNs and FNC (Braun et al., 2012; Tomasi et al., 2017; Van Dijk et al., 2010). In the case of our HCP analyses, our results closely relate to those reported by Tomasi et al., who evaluated reliability in the same dataset using various FC metrics and found between 4.5 and 12 min of data were required for stability, depending on the FC metric used for evaluation (Tomasi et al., 2017). Moreover, their finding that more data was required to achieve reliable estimates of ICA‐based spatial maps than was required for estimates of functional connectivity was also replicated in our results (Figure 7). This repeated finding indicates that higher order summaries of the data, such as FNC, are stable at even very short scan times and is especially impactful for development of FNC‐based classifiers, which we show can also perform well with relatively short scans, that can be used in clinical decision support.

**FIGURE 10:**
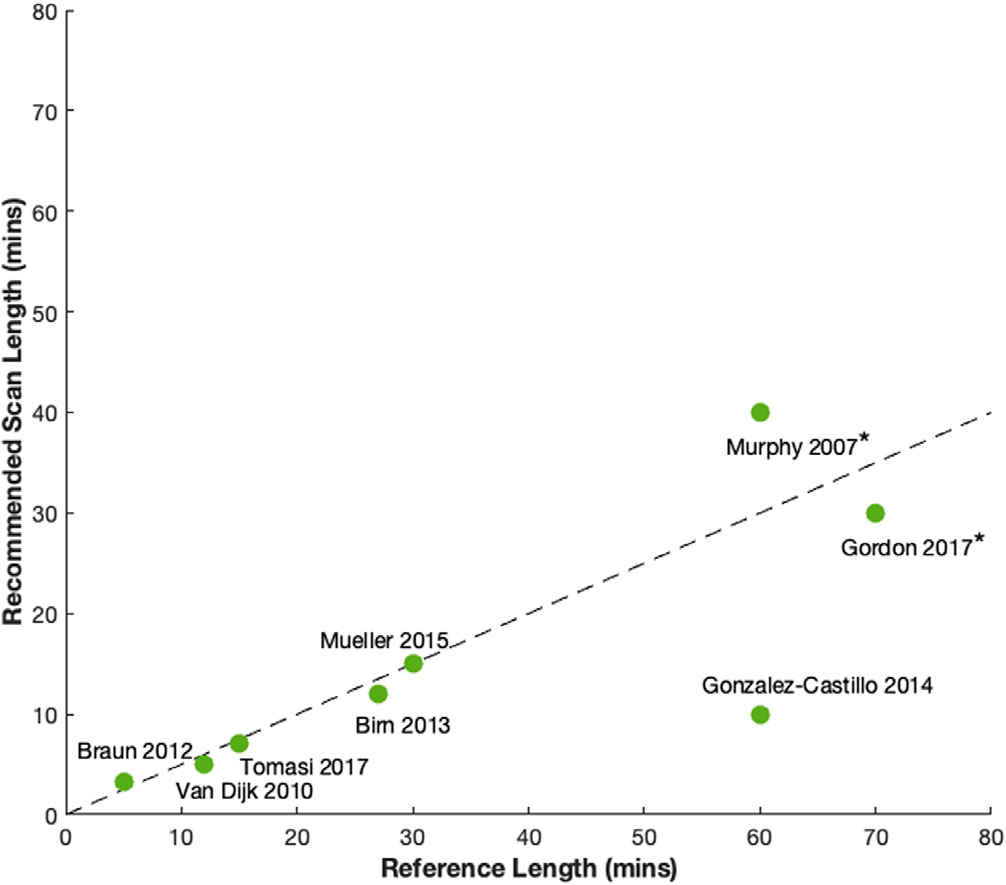
Summary of minimum recommended scan lengths as a function of the reference length used in recent studies of fMRI reliability. Dashed line shows trend ($y=\frac{x}{2}$). (*) denotes reference time series was comprised of two or more concatenated scans.

On the other hand, there are many studies which posit significantly longer scan times (10–30 min or more) are necessary to generate sufficiently reliable estimates of ICNs and connectivity. This connects to the second major theme of our results; the critical role reference length plays in studies of fMRI reliability. Figure 10 summarizes the findings of several recent studies of fMRI reliability, specifically minimum recommended scan length as a function of reference time course length. Though reliability is evaluated differently across this set of studies, the trend is clear: minimum reliable scan lengths are often recommended to be about half the length of the reference time course. Further, all studies that concluded minimum reliable scan lengths to be >10 min evaluated reliability against reference time courses >25 min (Birn et al., 2013; Gordon et al., 2017; Mueller et al., 2015; Murphy et al., 2007). These studies were all designed under the assumption that a prolonged rsfMRI scan (or the concatenation of several long rsfMRI time courses collected on different days) would provide a good “gold standard” approximation of individual rsFNC. However, considering the well‐documented increase of drowsiness, fatigue, and head motion as a function of scan time (Wang et al., 2017), it is unclear whether this assumption is correct. Our evaluation of the HCP data indicated that (1) the correspondence between ICN and FNC estimates from the first 5 min and last 5 min of the time series were low, (2) estimates generated from the first 5 min were more representative of the FNC and ICN estimates over the full range of scan lengths than those generated from the last 5 min, and (3) the positive linear trend in concordance of the “full” scan to the last 5 min reference estimate as a function of “full” scan length (Figure 8c,d) suggests the discrepancy between scan‐start and scan‐end estimates can more likely be attributed to increasing “scanner fatigue” effects common across all subjects rather than functional brain dynamics, which would be assumed to occur in unique patterns and at various timescales across subjects and therefore be averaged out in the group mean. This idea is further corroborated in our supplemental analyses that used FNC and ICN estimates from the last 1, 2, 3, and 4 min of scan time as the reference, which show poor concordance to corresponding estimates generated throughout the scan duration and is particularly evident in reliability of ICN estimates. The “optimal” amount of data for generating the most reliable ICN estimates is currently an open area of research, and our results align with other work in this space that suggest the highest reliability may be achieved when the tail end of the scan is not included (Iraji et al., 2022).

Relatedly, the final theme of our results is the consideration of the motivation or context within which reliability is being defined and studied. In the case of Gordon et al., as with many of the reliability studies discussed above, the goal was to identify the amount of data required to identify a reliable individual FNC “fingerprint” that was stable across a large amount of data, specifically across several scans collected over several days or weeks. The goal of our work was somewhat different, in that we sought to test how much a single short (or relatively long, in the case of the HCP analyses) scan could be further reduced and still provide reliable estimates of rsFNC and individual ICNs, specifically in the context of clinical utility and identification of group differences. Therefore, in addition to individual‐level reliability we also evaluated robustness of group‐level metrics as well as diagnostic classification performance, directly studying scan length reliability in the context of clinical utility. We found that even less data was required to reliably approximate significant group differences in rsFNC than at the subject level (2 min vs. 3.5 min in clinical experiments, 3 min vs. 4 min in simulated experiments), which is expected due to the benefit of group averaging. Surprisingly, we also found high reliability in classification performance at very short lengths, achieving a relative diagnostic accuracy of 97% compared to the full‐length reference TC with just 2 min of data. This result was somewhat unexpected, as classification of individuals is a much more difficult task than identifying group differences in aggregate. However, we found that even if overall rsFNC robustness was not reached with just 2 minutes of data, the rsFNC features that are generated at this very short scan length do indeed capture clinically relevant patterns of subject‐level rsFNC and yield diagnostic classifications that closely resemble those of the full reference TC.

This study is limited by the acquisition protocols of the clinical SZ data sets used in the analyses, which consist of relatively short (~5 min) one‐time scans of individuals. While HCP data was included for supplemental validation in longer and more temporally granular data, the nature of the healthy control cohort precluded the evaluation of the group analyses, which were the main interest of this work. We also note that while our results highlight important differences in rsFNC between individuals with SZ and controls that can be identified even in very short fMRI scans, the results should be interpreted with caution given the history of medication in the schizophrenia group. To address these limitations, one focus of future work might be collection of (1) slightly longer (~10 min vs. 5 min) fMRI scans in the clinical population to conduct a more robust evaluation of minimum clinical scan length, or (2) pre‐diagnostic/ pre‐intervention fMRI scans of those with suspected psychiatric conditions to ascertain the strength of possible medication effects with the added benefit of evaluation across a possible range of diagnoses. Furthermore, the results of the simulation study are expectedly limited in (1) the spatial dimensionality, simulating a single axial slice rather than a three‐dimensional full brain, (2) strength of engineered group differences, and (3) ability to accurately simulate the properties of true fMRI data. However, the efficacy of the SimTB software has been extensively studied and has shown utility for reliably modeling multi‐subject fMRI datasets (Allen et al., 2012; Erhardt et al., 2012). Additionally, we only consider the results of scICA with a single network template generated using only control subjects (Du et al., 2020). While most existing reliability studies consider only one brain atlas, a more thorough examination of scICA in the context of reliability and data reduction in the future could include various templates, such as customized templates for certain diagnostic groups or global templates generated with a higher model order (i.e., more granular spatial scale; Iraji et al., 2022). Finally, we acknowledge that the identification of a minimum scan length is dependent upon the metrics of interest, or the evaluation framework used. For example, as our work produced differing results for the evaluation of group‐level vs. subject‐level rsFNC reliability, future studies that examine other metrics, such as graph theoretic measures of rsFNC (i.e., modularity, global efficiency, etc.), could result in minimum scan recommendations that differ from those in this study.

Our results support the idea that when scICA is employed, a minimally sufficient scan length may exist in the 2–5 min range that could be favorable for use in clinical settings, both in maximizing clinical efficiency and patient comfort while retaining diagnostic efficacy. However, more work is still required to validate these results in larger (and longer) data sets, as well as in other diagnoses for which rsFNC biomarkers may be useful. Also, though we found short scans to show promise for clinical utility, we note that when possible, longer scan durations could be beneficial for maximizing information capture. Future work may also focus on the benefits of using scICA in the study of time‐resolved, or “dynamic” FNC (dFNC). The added regularization afforded by the spatial priors in scICA may help stabilize sliding window estimates of dFNC, potentially increasing the resolution at which dFNC can be studied with the use of smaller window sizes and increasing the reliability of dFNC results overall.

## 5 CONCLUSION

Due to the lack of consensus for minimum fMRI scan length recommendations, specifically for clinical biomarker applications, we studied the robustness of ICN and rsFNC measures with respect to scan length. We found just a fraction (2–7 min) of the full‐length time series was necessary to sufficiently approximate ICNs and rsFNC at both the subject‐ and group‐level. These findings were consistent across experiments in both shorter clinical data and longer simulated data. Overall, our results suggest clinical rsfMRI scans, when decomposed with scICA, could possibly be shortened to just 2–5 min without significant loss of rsFNC information or classification performance of longer scan lengths.

## Supporting information


**Figure S1.** Average ICN smoothness (computed as 1—normalized gradient) of original HCP data, HCP data with various levels of additional post‐hoc smoothing applied, and of the original FBIRN dataset. Post‐hoc smoothing at sigma = 0.5 (i.e., FWHM = 3.53 mm) in the HCP data yielded the closest match to the smoothness of FBIRN at the same data length, making for the fairest comparison between datasets. Thus, we selected the sigma = 0.5 smoothed dataset for our HCP comparative analyses.

**Figure S2.** Reliability results using the last 1–5 minutes of data as reference.

## Data Availability

Details on the availability of the FBIRN dataset used as our discovery dataset can be found at https://www.nitrc.org/projects/fbirn/. Details on the availability of the COBRE dataset used as our validation dataset can be found at http://fcon_1000.projects.nitrc.org/indi/retro/cobre.html. Details on the availability of the HCP dataset used in our additional validation experiments can be found at http://www.humanconnectomeproject.org/data/. The SimTB MATLAB toolbox used to generate our simulated data is freely available at https://trendscenter.org/trends/software/simtb/index.html. The code and network templates used for spatially constrained ICA are available at http://trendscenter.org/software.
