# Supplementary figures and images for: Reliability and clinical utility of spatially constrained estimates of intrinsic functional networks from very short fMRI scans
Source: Hum Brain Mapp. 2023 Feb 25;44(6):2620–35. doi: 10.1002/hbm.26234 (PMC10028646; doi:10.1002/hbm.26234)

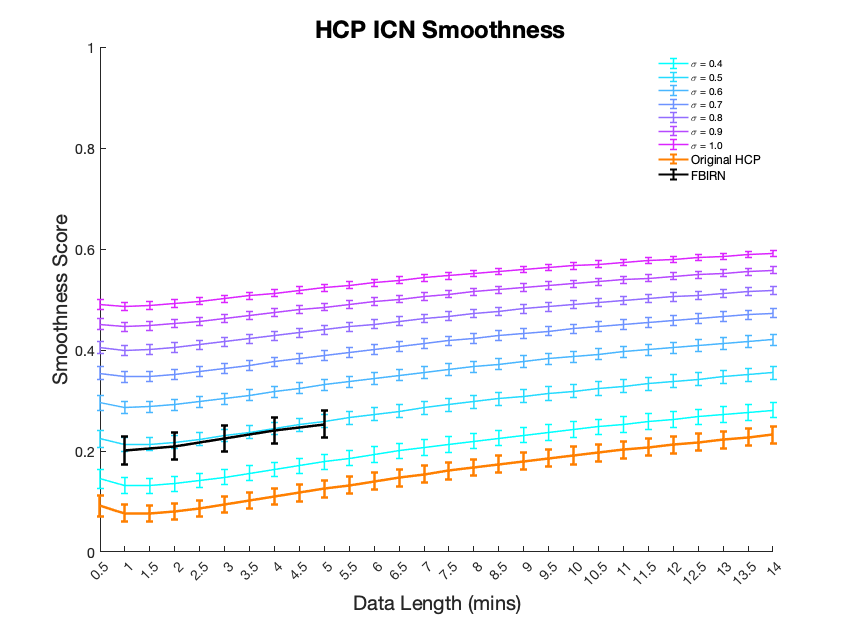

Supplement: Supplementary file 1 — Figure S1. Average ICN smoothness (computed as 1—normalized gradient) of original HCP data, HCP data with various levels of additional post‐hoc smoothing applied, and of the original FBIRN dataset. Post‐hoc smoothing at sigma = 0.5 (i.e., FWHM = 3.53 mm) in the HCP data yielded the closest match to the smoothness of FBIRN at the same data length, making for the fairest comparison between datasets. Thus, we selected the sigma = 0.5 smoothed dataset for our HCP comparative analyses. [file HBM-44-2620-s001.png]

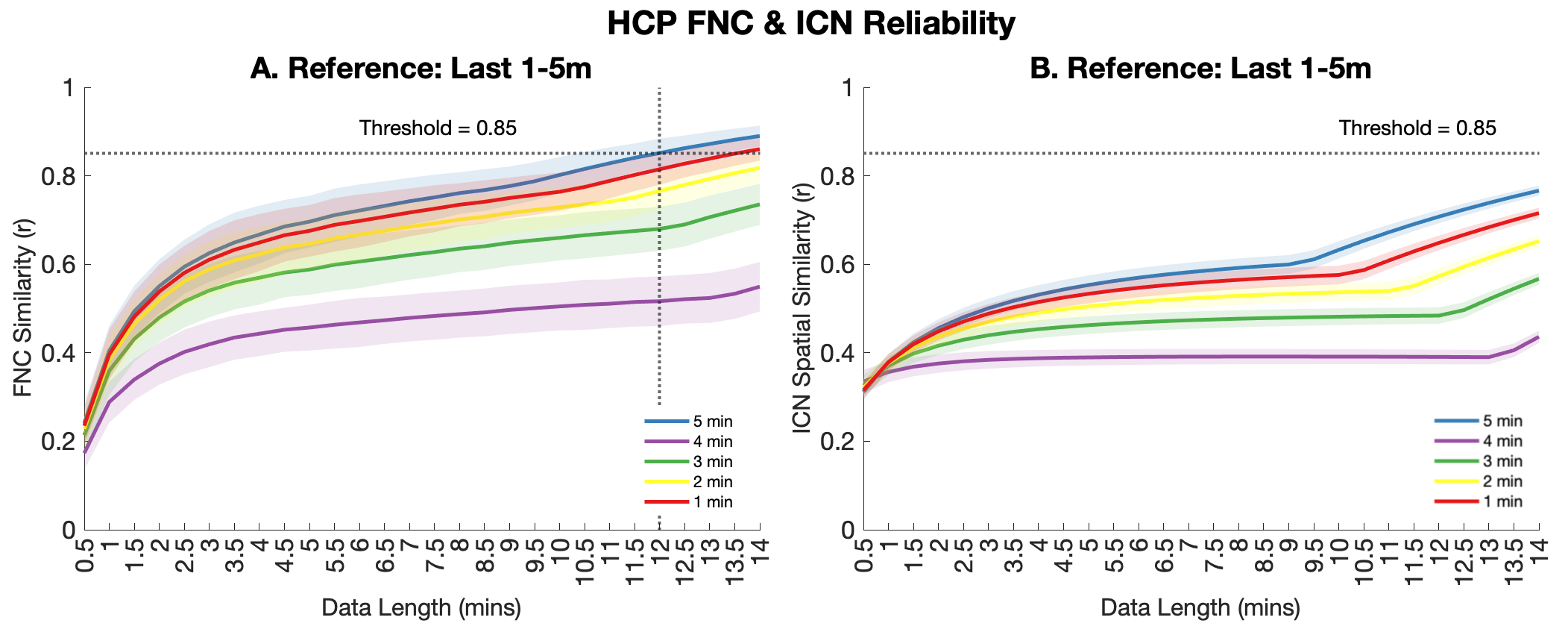

Supplement: Supplementary file 2 — Figure S2. Reliability results using the last 1–5 minutes of data as reference. [file HBM-44-2620-s002.png]
